# A Cross-Sectional Study on the Quality of Life of Caregivers of Children With Disability in Chennai, India

**DOI:** 10.7759/cureus.93178

**Published:** 2025-09-25

**Authors:** Swetha R S, Priya Pasupathy, Vijayakumar M

**Affiliations:** 1 Community Medicine, Coimbatore Medical College, Coimbatore, IND; 2 Community Medicine, Chengalpattu Medical College, Chengalpattu, IND; 3 Community Medicine, Government Medical College and Employees' State Insurance Corporation (ESIC) Hospital, Coimbatore, IND

**Keywords:** caregivers, children with disability, coping mechanism, depression, quality of life (qol)

## Abstract

Background

Disability influences a person's quality of life (QoL) in social, psychological, physical, and environmental dimensions. The caregivers of the disabled person equally share the burden, and their QoL is also greatly affected, as caregivers face substantial challenges in multiple dimensions. This limits their ability to focus their attention or time elsewhere, negatively impacting their social life and overall QoL. Consequently, understanding the QoL of caregivers, the variety of strategies they used to cope, and their mental health status, particularly depression, is crucial for providing support and intervention. Thus, this community-based study is carried out in Chennai, India, to assess the QoL, coping mechanisms, and depression among caregivers of children with disabilities.

Objectives

This study was done to assess the QoL of primary caregivers of children with disabilities, the coping strategies they use, and the prevalence of depression in them.

Methodology

This study was conducted as a community-based cross-sectional study in Chennai, India, among caregivers of children with disability. Participants were primary caregivers of children with disabilities for more than two years and provided informed consent. The paid caretaker was excluded from the study. A sample of 205 was recruited after multistage random sampling. Data was collected by in-person interview using a semi-structured pretested questionnaire and also included the World Health Organization Quality of Life-BREF (WHOQOL-BREF) (total score range: 0-400), the Brief Coping Orientation to Problems Experienced (Brief-COPE) (score: 2-8), and the Center for Epidemiologic Studies Depression Scale-Revised 10-item (CESD-R10) (range: 0-30). The data obtained were entered in Microsoft Excel (Microsoft Corporation, Redmond, Washington, United States) and analyzed using IBM SPSS Statistics for Windows, Version 16.0 (SPSS Inc., Chicago, Illinois, United States). The chi-squared test was used for categorical associations, while analysis of variance (ANOVA) was used to compare group means.

Results

Among 205 caregivers, 85.9% were female, with mothers comprising 80%. Children's mean age was 8.64±4.16 years. Motor delay (26.3%) was the most common disability. The mean WHOQOL-BREF score was 170.96±59.07, and 39% of caregivers reported average QoL, both reflecting moderate subjective well-being. QoL differed significantly across disability types (F=6.57; p<0.001) and was associated with type of disability (p=0.001), number of siblings (p=0.038), and socioeconomic status (p=0.004). Emotion-focused coping (4.56±0.83) was most common, followed by problem-focused (4.05±1.16) and avoidant strategies (3.42±0.74). Depression prevalence was 33.2%, significantly associated with caregiver-child relationship (p=0.016), caregiver age (p=0.012), child's age (p=0.042), mother's occupation (p=0.042), and family size (p=0.017).

Conclusion

Caregivers of children with disabilities in Chennai have a markedly lower QoL, especially concerning environmental and physical health aspects. Caregivers mostly used the emotion-focused religious and acceptance strategies. One-third of them had a risk for depression. These findings underscore the dual burden faced by family caregivers and highlight the urgent need for comprehensive measures to support affected families. Empowering caregivers by providing psychosocial support, community integration, and financial assistance is vital.

## Introduction

The International Classification of Functioning, Disability and Health (ICF) defines disability as "an umbrella term for impairments, activity limitations and participation restrictions. It denotes the negative aspects of the interaction between an individual (with a health condition) and that individual's contextual factors (environmental and personal factors)" [1]. Disability influences a person's quality of life (QoL) in social, psychological, and environmental dimensions [2]. The caregivers of the disabled child equally share the burden of disability, and their QoL is also greatly affected.

In the Indian context, the individual with a disability is typically cared for by family members. Due to the focus on the person with a disability, the emotional and physical health of the caregiver is often neglected. Most carers' time is spent caring for the child with serious developmental delays and impairments. This limits their ability to focus their attention or time elsewhere, which in turn negatively affects their social life and overall QoL. They experience varied difficulties, including disruptions in routine, family leisure time, family health, work absenteeism, physical and mental stress, and higher financial costs [3,4].

Compared to the general population, caregivers of people with impairments have been found to have a significantly worse QoL. A heavier caregiver workload translates to a lower QoL [5]. The emotional and physical experiences involved in providing care can strain even the most capable person, resulting in feelings of anger, anxiety, sadness, isolation, exhaustion, and then guilt for having these feelings [6]. Five characteristics that have been demonstrated to be associated with poor health outcomes for caregivers are the severity of the disability, the child's behavior, the child's temperament, the caregiver's low self-esteem, and the caregiver's lack of social support [7].

The burden of disease is, however, perceived differently by each caregiver due to differences in their methods of coping. Coping is the use of cognitive and behavioral strategies to overcome challenges faced by caregivers [8]. Coping will lessen the potential stress that these issues may generate.

This study aims to assess the QoL of caregivers of children with disabilities across physical, psychological, social, and environmental domains. It also seeks to identify the coping strategies employed by caregivers and determine the prevalence of depressive symptoms.

By focusing on these objectives, the study addresses an important evidence gap, as research on caregivers of children with disabilities is limited in developing countries, particularly in India.

## Materials and methods

Study design and setting

This community-based cross-sectional study was conducted among primary caregivers of disabled children residing in three zones of the Greater Chennai Corporation, Tamil Nadu, India.

Study population and sampling

The study was conducted among familial caregivers of children with disabilities residing in selected zones of the Greater Chennai Corporation, Tamil Nadu, India. Participants were recruited through a multistage sampling technique. The central region of the Greater Chennai Corporation was chosen by simple random sampling out of the three regions (northern, southern, and central). The central region has five administrative zones (6-10), and among them, zones 6, 7, and 8 were selected by the lottery method.

A line list of all children under 18 years with registered disabilities in the zones was obtained from the records. Eligible households were then randomly selected using a computer-generated random number table from this sampling frame until the required sample size was attained. Non-responding households were replaced by the next randomly selected household.

The sample size was calculated using the formula $\text{n}=\left(\frac{\text{Z&alpha;}}{2}\times\frac{\text{SD}}{\text{d}}\right)^{2}$, with a standard deviation of 20.15, based on a previous study [9], Z equal to 1.96 (for 95% CI), and the precision set at 3. Hence, $\left(\frac{1.96\times20.15}{3}\right)^{2}=174$. Another 15% for non-response was added and rounded off to 210 samples. Eligible caregivers were approached, of whom 205 consented and completed the interview. Caregivers who had lived with and primarily cared for a child with a disability for over two years were considered eligible. Caregivers were defined as family members directly involved in the child's daily activities. Paid or professional caregivers were excluded.

Data collection and study tool

Primary data were collected through in-person interviews at caregivers' homes using a pretested semi-structured questionnaire. Instruments were translated into the local language (Tamil) using forward and backward translation by bilingual experts. Content validity was assessed by a panel of subject experts, and a pilot test was conducted. To minimize interviewer bias, all interviews were conducted by trained investigators in the local language following a standardized protocol. The questionnaire comprised four sections. The first section recorded sociodemographic details of both the caregiver and the child, such as age, gender, education, occupation, family structure, and socioeconomic status. The second section focused on the QoL assessed by the World Health Organization Quality of Life-BREF (WHOQOL-BREF) [10]. It comprises 26 items grouped into four domains: physical health, psychological health, social relationships, and environment. Items are scored on a 5-point Likert scale and transformed to a 0-100 scale in each domain, summing up to 400. Higher scores indicated better QoL. In addition, we also considered responses to the first global item ("How would you rate your QoL?", rated from 1 (very poor) to 5 (very good)) to categorize subjective QoL as very poor, poor, neither poor nor good, good, or very good. For the chi-squared test, the good or very good categories were grouped as good QoL, while the remaining categories were combined as poor QoL. The permission to use the tool was obtained. The third section assessed the coping strategies by utilizing the Brief Coping Orientation to Problems Experienced (Brief-COPE) scale [11]. This evaluates 14 coping strategies, each measured by 2 items (range 2-8 per scale). Responses are rated on a 4-point Likert scale (1=never; 4=always), with higher scores indicating greater use of that strategy. For analysis, the 14 subscales were grouped into three categories, namely, problem-focused, emotion-focused, and avoidant coping strategies, with each category ranging from 2 to 8. The fourth section evaluated depressive symptoms using the Center for Epidemiologic Studies Depression Scale-Revised 10-item (CESD-R10) [12]. The score consisted of a 4-point Likert scale (0-3) with a total score range of 0-30, and a cutoff score of ≥10 was used to indicate clinically relevant depressive symptoms, consistent with prior research.

All study instruments (WHOQOL-BREF, Brief-COPE, CESD-R10) are freely available for use in academic and non-commercial research. 

Ethical consideration

Ethical approval was obtained from the Institutional Ethics Committee of Madras Medical College (approval number: 32102019). Official permission to conduct the study was obtained from the Deputy Commissioner (Health), Greater Chennai Corporation, Tamil Nadu, India. Informed written consents were obtained from the participants in the local language (Tamil). Participation in the interview was voluntary, and confidentiality was maintained.

Data analysis 

Data was entered into Microsoft Excel Version 2019 (Microsoft Corporation, Redmond, Washington, United States) and thoroughly checked for completeness and accuracy. Statistical analyses were performed using IBM SPSS Statistics for Windows, Version 16.0 (SPSS Inc., Chicago, Illinois, United States).

Data were summarized using descriptive statistics. Continuous variables were presented as means and standard deviations, whereas categorical variables were expressed as frequencies and percentages. The chi-squared test was used in analyzing the association between the categorical variables. One-way analysis of variance (ANOVA) was used to compare the means between the disability groups. A p-value of <0.05 was considered statistically significant.

## Results

Sociodemographic details

The study included 205 caregivers of children with disability, predominantly female (85.9%). Among the disabled children, 60% were boys. Caregivers' ages ranged from 22 to 65 years (mean 36.32±9.5; median 34). Most caregivers were aged 30-39 years (53.2%). Children's age ranged 1-18 years (mean 8.64±4.16; median 8) with the largest group being those aged 6-10 years (34.6%), followed by 11-15 years (30.2%) and 0-5 years (28.3%). The primary caregiver was most often the mother (80%), followed by the father (10.7%) and other relatives (9.3%).

Table 1 summarizes the distribution of disability in children. Motor delay was the most prevalent disability (26.3%), followed by mental retardation (14.6%) and learning disability (14.1%), while vision impairment was the least common at 1%.

**Table 1:** Distribution of disability type in children (n=205)

Disability type	Frequency (percentage)
Motor delay	54 (26.3%)
Mental retardation	30 (14.6%)
Learning disability	29 (14.1%)
Autism	20 (9.8%)
Hearing impairment	20 (9.8%)
Speech impairment	19 (9.3%)
Multiple disabilities	16 (7.8%)
Attention-deficit/hyperactivity disorder	15 (7.3%)
Vision impairment	2 (1%)

Concerning parental education and occupation, 42.9% of fathers and 27.9% of mothers were graduates, while 54.1% of the mothers were homemakers. Most families were classified as belonging to the lower middle (36.1%) or middle class (30.2%).

QoL

The average total WHOQOL-BREF score was 170.96 (SD 59.07; range 50.89-396.35). On the global QoL item, 39% of caregivers rated their QoL as neither poor nor good, while 30.2% rated it as poor and 10.7% as very poor (Table 2).

**Table 2:** Self-rated quality of life by caregivers using WHOQOL-BREF (n=205) WHOQOL-BREF: World Health Organization Quality of Life-BREF [10]

Self-rate of quality of life	Frequency (percentage)
Very poor	22 (10.7%)
Poor	62 (30.2%)
Neither good nor poor	80 (29%)
Good	36 (17.6%)
Very good	5 (2.4%)

Based on WHOQOL-BREF domains, the social relationships domain score was highest (44.3±19.7), while the environment domain score was found to be lowest (39.5±18.1) (Table 3).

**Table 3:** Domain-wise WHOQOL-BREF scores WHOQOL-BREF: World Health Organization Quality of Life-BREF [10]

Domain	Minimum	Maximum	Median	Interquartile range (25th-75th percentile)
Environment	6.25	93.75	37.50	31.25-50.00
Social relationships	8.33	100.00	41.66	33.33-58.33
Physical health	3.57	96.43	39.28	31.25-53.57
Psychological health	9.60	96.50	41.66	41.67-54.17

Caregivers of children with both speech impairment and attention-deficit/hyperactivity disorder (ADHD) reported the highest mean QoL scores, and the lowest score was observed in caregivers of children with multiple disabilities. ANOVA analysis (Table 4) showed that QoL scores differed significantly between the disability groups. Subsequent post hoc testing revealed that caregivers of children with speech impairment had significantly higher QoL scores than those caring for children with autism, mental retardation, multiple disabilities, and motor delay. Caregivers for children with multiple disabilities had significantly lower scores compared to those caring for children with hearing impairment, learning disabilities, ADHD, and motor delays.

**Table 4:** Comparison of total QoL scores across the disability types The one-way ANOVA test is used. A p-value of less than 0.05 is considered statistically significant. The WHOQOL-BREF [10] is used. df=204; F=6.570; p<0.0001 QoL: quality of life; ANOVA: analysis of variance; WHOQOL-BREF: World Health Organization Quality of Life-BREF

Disability type	Physical health domain (mean±SD)	Psychological health domain (mean±SD)	Social relationships domain (mean±SD)	Environment domain (mean±SD)	Total mean±SD
Autism	34.86±9.97	44.44±16.04	38.88±19.24	36.60±19.24	154.80±57.46
Mental retardation	30.95±18.07	47.22±15.09	37.77±18.40	35.31±16.27	151.26±54.57
Hearing impairment	46.42±11.52	45.00±10.43	47.08±13.85	42.18±11.49	180.69±29.38
Speech impairment	52.32±20.76	53.12±15.69	61.66±26.40	54.37±22.61	221.48±79.19
Vision impairment	23.21±22.72	35.41±26.51	29.16±5.89	50.00±17.67	137.79±37.45
Attention-deficit/hyperactivity disorder	56.19±14.99	50.83±6.71	52.22±8.60	52.08±15.52	211.32±6.30
Learning disorder	42.85±14.39	54.16±9.95	41.66±14.47	40.62±15.96	176.48±39.58
Motor delay	39.74±18.07	47.91±10.76	41.97±21.28	38.07±15.14	167.71±54.92
Multiple disorder	21.65±17.71	34.11±16.82	36.45±16.63	21.09±17.13	113.31±60.06

Association of QoL with demographic factors

As presented in Table 5, type of disability (p=0.001), number of siblings (p=0.038), and socioeconomic class (p=0.004) were significantly associated with QoL. Most caregivers of children with autism (95%) reported poor QoL, while the majority of those caring for children with motor delay (88.9%) reported good QoL.

**Table 5:** Association between QoL and selected demographic factors The chi-squared test is used. *A p-value of <0.05 is considered statistically significant. QoL: quality of life

Characteristic	Category	Good QoL n (%)	Poor QoL n (%)	Chi-squared value	P-value
Age (years)	20-29	9 (20.9)	34 (79.1)	1.57	0.81
	30-39	19 (17.4)	90 (82.6)		
	40-49	7 (22.6)	24 (77.4)		
	50-59	4 (30.8)	9 (69.2)		
	≥60	2 (22.2)	7 (77.8)		
Gender	Male	5 (17.2)	24 (82.8)	0.16	0.69
	Female	36 (20.5)	140 (79.5)		
Siblings	None	8 (11.8)	60 (88.2)	5.79	0.038*
	≥1	33 (24.1)	104 (75.9)		
Socioeconomic status	Upper	31 (27.2)	83 (72.8)	8.30	0.004*
	Lower	10 (11)	81 (89)		

Coping strategies

On using the Brief-COPE scale, emotion-focused coping was the most commonly used strategy, followed by problem-focused and avoidant coping, as depicted in Table 6. The most frequently used individual coping styles were religion, emotional support, venting, and acceptance, while denial and substance use were the least used.

**Table 6:** Distribution of coping strategies The Brief-COPE scale [11] is used. Brief-COPE: Brief Coping Orientation to Problems Experienced

Type of disability	Emotional focus (mean±SD)	Problem focus (mean±SD)	Avoidant focus (mean±SD)
Autism	4.74±0.74	4.39±1.12	3.31±0.70
Mental retardation	4.3±0.98	3.77±1.26	3.22±0.71
Hearing impairment	4.5±0.97	4.11±0.87	3.59±0.59
Speech impairment	4.91±0.92	5.00±1.32	3.58±0.78
Vision impairment	4.00±0.23	3.25±0.34	3.5±1.41
Attention-deficit/hyperactivity disorder	4.93±0.65	4.07±0.74	3.75±0.83
Learning disorder	4.55±0.60	4.12±1.12	3.44±0.81
Motor delay	4.44±0.78	3.84±1.09	3.38±0.79
Multiple disorder	4.71±0.87	3.59±1.47	3.31±0.48

Depression among caregivers

The average score on depression (CESD-R10) was 10.8±4.4, among which 33.2% screened positive for depression. Depression was more common among caregivers aged 20-29 years (46.5%) and grandparents (57.9%). Depression was significantly associated with the child's age (p=0.042), the mother's occupation (p=0.042), and family size (p=0.017) as shown in Table 7.

**Table 7:** Association of depression and demographic factors The chi-squared test is used. *A p-value of <0.05 is considered statistically significant. The CESD-R10 [12] is used. CESD-R10: Center for Epidemiologic Studies Depression Scale-Revised 10-item

Characteristic	Category	Depressed n (%)	Not depressed n (%)	Chi-squared value	P-value
Age (years)	20-29	20 (46.5)	23 (53.5)	12.92	0.012*
	30-39	30 (27.5)	79 (72.5)		
	40-49	6 (19.4)	25 (80.6)		
	50-59	5 (38.5)	8 (61.5)		
	≥60	4 (55.6)	4 (44.4)		
Gender	Male	8 (27.6)	21 (72.4)	0.47	0.49
	Female	60 (34.1)	116 (65.9)		
Relationship	Parent	57 (30.6)	129 (69.4)	5.8	0.016*
	Grandparent	11 (57.9)	8 (42.1)		
Child's age	0-8 years	43 (39.4)	66 (60.6)	4.14	0.042*
	8-18 years	25 (26)	71 (74)		
Mother's occupation	Unemployed	30 (27)	81 (73)	4.12	0.042*
	Employed	38 (40.4)	56 (59.6)		
Family size	≤4	43 (28.5)	108 (71.5)	5.7	0.017*
	>4	25 (46.3)	29 (53.7)		

## Discussion

A cross-sectional study was conducted in three zones of the Greater Chennai Corporation to assess the QoL, coping strategies, and prevalence of depression among caregivers of children with disabilities. Utilizing the WHOQOL-BREF, Brief-COPE, and CESD-R10 instruments, it was found that most caregivers had poor or very poor QoL. They predominantly used emotion-focused strategies to cope with the caregiving role, and approximately one-third had depressive symptoms.

Most of our study participants were mothers, which is consistent with other studies conducted in India [13,14]. Women in India often bear the burden of caregiving responsibilities. This pattern is consistent with cultural expectations and places mothers under elevated psychosocial pressure. Most children with disabilities were boys, as reported in the earlier studies from Gujarat [9].

Overall QoL scores were low, with the environment domain scoring worst. This reflects the struggle for financial stability, healthcare access, and leisure opportunities that parents of disabled children face. Studies from Gujarat and Mysore reported similar findings [9,15]. However, one study noted slightly better scores in either the psychological or social domains [13]. The differences in global settings could also be attributed to the level of social support, service availability, and cultural practices. Notably, QoL had a significant correlation with socioeconomic status, type of disability, and number of children. Caregivers of children with multiple disabilities had the worst QoL, indicating the heavy burden these parents experience. These findings are also consistent with studies showing that poverty and severity of disability influence the well-being of caregivers [13,15].

The major coping strategies used by caregivers in our study were emotion-focused, specifically religious practices, acceptance, and emotional support. While these strategies might offer temporary relief, they can be maladaptive in the long run. Studies conducted by Ganjiwale et al. [9] in Gujarat and Long et al. [16] in Vietnam also revealed emotional coping as a frequently used strategy by the caregivers.

In contrast, problem-focused coping was the least utilized strategy despite studies indicating that structured problem-solving and planning reduced caregiver stress [17]. This calls for the development of psychosocial interventions that seek to reinforce adaptive coping behaviors.

In the current study, about one-third of the caregivers experienced depressive symptoms. This study finding is similar to a systematic review by Scherer et al., which reported a prevalence of 31% among parents of children with disabilities [18]. In contrast, Dave et al. [19], in a study conducted in Gujarat, reported a significantly higher caregiver prevalence of 63%. Note that their study was limited to caregivers of children with intellectual disabilities, while ours involved the caregivers of children with all forms of disabilities, which might suggest that the type of disability affects the risk.

Depression had an association with caregiver age, child's age, caregiver relationship to the child, maternal employment status, and family size. Mothers and grandparent caregivers appeared to be particularly at risk because of their multiple work roles. These findings underscore the interplay between demographic and caregiving factors in determining women's mental health.

The findings have significant implications. There is a need to screen caregivers and identify their specific needs. Services such as counseling and peer support groups should be readily accessible. Policies should focus on empowering caregivers by addressing their socioeconomic needs. 

Since most caregivers employ emotion-based coping strategies, it is vital to implement measures that focus on stress alleviation. Some of the intervention measures include community-based peer support systems in conjunction with counseling workshops focusing on stress management. Linking these caregivers to local non-governmental organizations (NGOs) and social welfare programs would also provide financial aid and psychosocial support. 

The study has several strengths, including a relatively large sample size and the use of standardized and validated tools. However, the study is limited by its design. The cross-sectional nature of the study limits causal inferences. Since our study was conducted in an urban setting, the findings may not be generalizable to caregivers in rural areas, which constitute a large proportion of India's population. Future research should focus on rural caregivers to better understand their experiences and challenges.

## Conclusions

This study shows that the caregivers of children with disabilities in urban Chennai have poor QoL, predominantly use emotion-focused coping strategies, and have a high prevalence of depressive symptoms. Mothers and grandparents seem especially at risk since they have multiple caregiving roles and work responsibilities. These findings underscore the importance of addressing caregivers' mental health needs. Future studies need to examine the efficacy of specific interventions, such as structured programs for coping skills or community support, and investigate causal links between caregiving burden and depression.
